# Analysis of the relationship between community characteristics and depression using geographically weighted regression

**DOI:** 10.4178/epih.e2017025

**Published:** 2017-06-21

**Authors:** Hyungyun Choi, Ho Kim

**Affiliations:** 1 Korea Centers for Disease Control and Prevention, Cheongju, Korea; 2 Graduate School of Public Health, Seoul National University, Seoul, Korea; 3 Asian Institute for Energy, Environment and Sustainability, Seoul, Korea

**Keywords:** Depression, Depressive disorder, Spatial regression, Spatial analysis, Health status

## Abstract

**OBJECTIVES:**

Achieving national health equity is currently a pressing issue. Large regional variations in the health determinants are observed. Depression, one of the most common mental disorders, has large variations in incidence among different populations, and thus must be regionally analyzed. The present study aimed at analyzing regional disparities in depressive symptoms and identifying the health determinants that require regional interventions.

**METHODS:**

Using health indicators of depression in the Korea Community Health Survey 2011 and 2013, the Moran’s I was calculated for each variable to assess spatial autocorrelation, and a validated geographically weighted regression analysis using ArcGIS version 10.1 of different domains: health behavior, morbidity, and the social and physical environments were created, and the final model included a combination of significant variables in these models.

**RESULTS:**

In the health behavior domain, the weekly breakfast intake frequency of 1-2 times was the most significantly correlated with depression in all regions, followed by exposure to secondhand smoke and the level of perceived stress in some regions. In the morbidity domain, the rate of lifetime diagnosis of myocardial infarction was the most significantly correlated with depression. In the social and physical environment domain, the trust environment within the local community was highly correlated with depression, showing that lower the level of trust, higher was the level of depression. A final model was constructed and analyzed using highly influential variables from each domain. The models were divided into two groups according to the significance of correlation of each variable with the experience of depression symptoms.

**CONCLUSIONS:**

The indicators of the regional health status are significantly associated with the incidence of depressive symptoms within a region. The significance of this correlation varied across regions.

## INTRODUCTION

Achieving health equity is a pressing current issue. It involves resolving health inequalities, which are the differences in health status between different populations [1]. It can ultimately increase the national lifespan. An example of health inequalities is the differences in health status among populations of different regions [2]. Large variations in health indicators and health determinants are seen across regions [3,4], yet research on the factors associated with the regional health status is rare. While research results that consider these factors have been published, most studies have dealt with chronic diseases [5-9]. While chronic diseases have received much attention, and have been consistently managed nationally, mental disorders have often been overlooked as being unimportant and have not been actively managed despite the fact them being an issue that not only affects individuals, but the society as a whole [10]. Incidence of depression, one of the most common mental disorders, has shown large regional variations among different populations [11,12], necessitating research on its regional prevalence.

Depression has previously been reported to damage an individual’s social and physical peace more significantly than chronic diseases [13], and negatively affect family relationships and social life, compromising the overall quality of life, and causing suicide in 10-15% of patients that it affects [14]. The rate of suicide in the same year was 27.3 per 100,000 persons in Korea, more than twice that of the mean rate of suicide of the Organization for Economic Cooperation and Development countries. In a study on global burden of diseases, major depressive disorder was ranked 15th among 291 diseases in terms of disability adjusted life years (DALY) [15], and moved to the 11th position in 2010. The DALY is the sum of years of life lost and years lived with disability (YLD). The YLD of Korea was ranked 3rd in 1990, and 2nd in 2013, indicating that while depression is not a critical cause of death, it can significantly negatively affect an individual’s well-being. Not only is depression a major cause of suicide, but it can also produce other negative results. Therefore, depression must be managed to improve the nation’s quality of life and for the sake of long-term national development.

Depression is associated with individuals’ biological vulnerability (age and sex) [16,17], social capital [18,19] including participation in social activities [20] and environment of trust in a community [21,22], and various health behaviors [10] such as having a chronic disease [23,24], smoking [25], drinking, exercising, and sleeping hours. Large variations in the distribution of these factors that affect depression have been observed [26], suggesting that there may be regional differences in the prevalence of depression. Gaining insights into these factors, which can be controlled through an intervention, is a meaningful approach toward health promotion.

In the present study, we aim to identify health determinants that require regional intervention by analyzing regional variations of depression and propose a methodology and method of presentation of research results that can be used in future research on regional disparities in health status.

## MATERIALS AND METHODS

### Materials

To investigate the associations between regional characteristics and depression, the latest data from the Korea Community Health Surveys (KCHS) for each administrative district in 2013 were used. However, considering the possibility of reverse causation between the health behavior variables and the dependent variables due to the nature of the topic in this study, the data in 2013 was used for the dependent variables, and for the independent variables, the mean value of the data in 2013 and 2011 was used as some data had been collected in every two years. Considering that repeated cross-sectional survey data consisting of independent samples in each year were used, there is a possibility of errors in determining the sample size and conducting the survey [7], which would require adjustments, and it was determined that data obtained from multiple years were more appropriate than those from a single year since regional health promotion programs are often in the form of a long-term intervention.

The lifetime diagnosis of depression and the rate of experiencing depressive symptoms are some of the indicators of depression investigated in the KCHS. The lifetime diagnosis of depression is dependent on the physicians’ diagnoses, and is a measure of the number of cases of confirmed illness. However, it only applies to cases where patients visit a hospital or clinic on their own to receive the diagnosis, such that, considering the nature of mental disorders, its prevalence more likely to be underestimated than that of other diseases, is higher than the diagnosis of other diseases, and is associated with many confounding factors. Conversely, as an indicator of the underlying emotions of depression, the rate of experiencing depressive symptoms is not necessarily diagnosed by a physician, but based on responses to the questionnaire question, “Have you felt sadness or despair that continuously interfered with your daily life for more than two weeks in the last one year?”, which can actually reflect some diagnostic criteria of depression, and thus, has been selected as a dependent variable to explain depression. The Diagnostic and Statistical Manual of Mental Disorders of the American Psychiatric Association defines depression as when five or more of the following symptoms continue for two weeks, and changes in functional ability occur: a continuous feeling of depression throughout an entire day, loss of all kinds of joy and interest, significant weight loss due to loss of appetite, insomnia or hypersomnia that continue for days, psychomotor changes, fatigue or loss of energy, a feeling of worthlessness or unreasonable guilt, impaired thinking, concentration and judgment, and repeated suicidal ideation and planning [27].

After reviewing prior study associated with depression, indicators were selected as the independent variables. Indicators highly correlated with the dependent variables were subsequently selected.

A total of 50 available variables including the dependent variables were finally selected, and a total of 215 administrative districts were targeted for the study by adjusting the definition of a district based on the criteria in 2013 if there was a discrepancy in the number of administrative districts between the data of 2011 and 2013 due to the merging of administrative districts during the period.

### Statistical analysis

All indicators were directly standardized by sex after dividing the subjects into age groups with an age gap of 10 years for accurate comparisons between regions.

A correlation analysis was performed to verify the association between the indicators that were determined to be associated with depression based on literature review and the rate of experiencing depressive symptoms. Variables with an absolute value greater than or equal to 0.2 at the level of significance of 5% were subjected to spatial analysis. Of the variables whose correlation with depressive symptoms was below the significance level, those with a possibility for an intervention or were considered to be quite important in healthcare were included in the analysis.

The data of administrative areas were used in this study. Because the effects of the factors that were actually associated with depression could be reduced by the arbitrary classification of administrative areas, physical proximity between the areas had to be considered. Accordingly, we checked if regions of similar degrees of variations were adjacent to one another by calculating Moran’s I to investigate spatial patterns measured with an interval scale. After confirming spatial autocorrelation based on Moran’s I, we performed a geographically weighted regression analysis (GWR). The adequacy of a model is affected by the bandwidth settings. A fixed kernel is used for samples showing regular patterns of distribution, and an adaptive kernel is used for samples with irregular patterns of distribution to increase the adequacy of the model [28]. In this study, because the distances between areas were not uniform, small bandwidths had to be used for areas close to one another, and large bandwidths for areas relatively far from each other. For this, we used an adaptive kernel to set the bandwidth and an Akaike’s information criterion (AIC), which is generally easy to use, to assess the adequacy of our models.

The GWR model consisted of three domains. The variables of each domain were analyzed, and those found to be statistically significantly correlated with depressive symptoms were used to construct a final model. This was to reduce bias for each administrative area since the number of primary variables included in the model is limited by the strict criteria for multicollinearity. All statistical analyses were performed using ArcGIS version 10.1 (ESRI, Redlands, CA, USA).

To identify the variables with significant influences in each region among the variables found to be associated with depressive symptoms in the GWR analysis, regression coefficients for all administrative areas were converted to Z-scores and compared. The Z-score is a standardized score, and the deviation score from the mean score were divided by the standard deviation of the distribution for relative comparison. The Z-score was calculated for each variable for all administrative areas, and ranked to identify the variables with the highest ranking in each area.

## RESULTS

### Regional differences in the depression-associated factors

In case of extreme expenses to identify regional differences, The rate of performing moderate to high intensity physical activities, The rate of consulting a psychologist due to stress, The rate of lifetime diagnosis of atopic dermatitis and allergic rhinitis were high (Table 1).

### Spatial correlation of cluster types by region

Thirty three variables were found to be significantly correlated with the rate of experiencing depressive symptoms at a significance level of 5% in a correlational analysis. The rate of lifetime diagnosis of allergic rhinitis was the most correlated with that of feeling depressed with a correlation coefficient of 0.65 (Table 2).

Most variables selected from the correlation analysis were significantly positively autocorrelated at a significance level of 5% in the spatial autocorrelation analysis, and were deemed adequate for a GWR analysis. The rate of experiencing depressive symptoms, which was a dependent variable, had a Moran’s I value of 0.34, and exhibited clustering (Table 2).

### Community characteristics and feelings of depression

To investigate the factors that are closely associated with depressive symptoms among various factors of health science, we constructed the final model shown below. The variables in the equation were written in the order of the highest correlation with the rate of depressive symptoms.

Y (rate of experiencing depressive symptoms)
=B0+B1 (rate of diagnosis of myocardial infarction)
+B2 (rate of lifetime allergic rhinitis)
+B3 (weekly breakfast intake frequency of 1-2 times)
+B4 (perceived stress level)
+B5 (environment of trust in community)+ε

**Figure 1.**
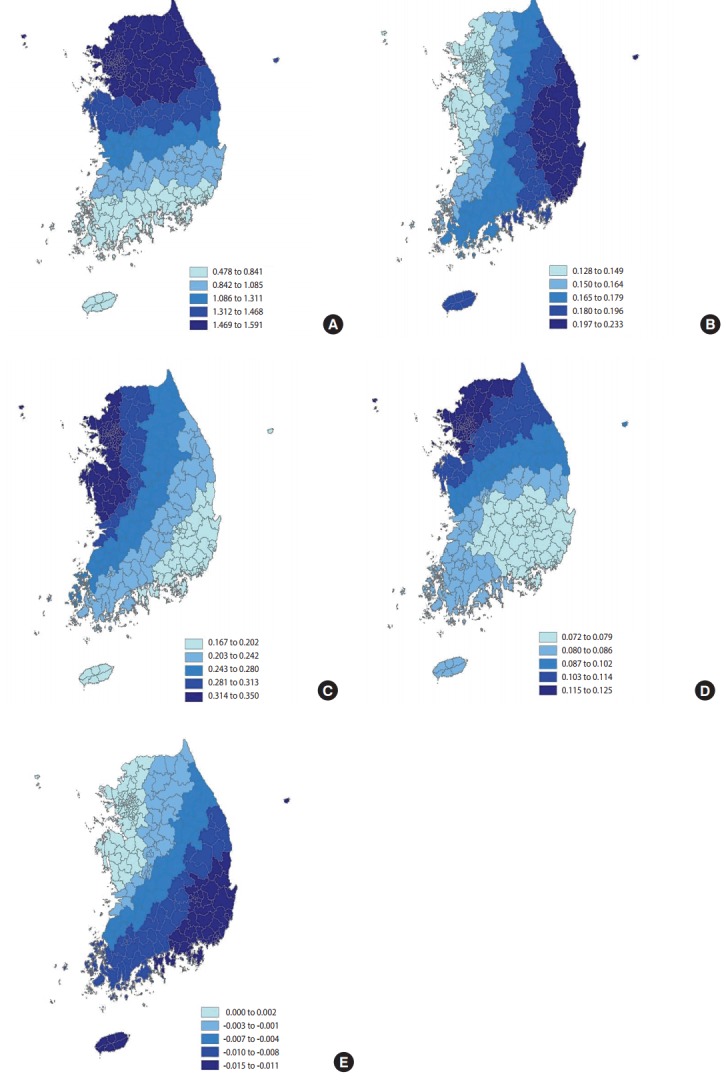
Regression coefficient based on geographically weighted regression (A) rate of lifetime diagnosis of myocardial infarction, (B) rate of lifetime diagnosis of allergic rhinitis, (C) weekly breakfast intake frequency of 1-2 times, (D) perceived stress level, and (E) public transit environment.

The five variables included in the model were all significantly correlated with the rate of experiencing depressive symptoms. The rate of lifetime diagnosis of myocardial infarction was the most correlated. In some areas, the rate of lifetime diagnosis of myocardial infarction increased by 1%, and that of experiencing depressive symptoms increased by 1.21% (p= 0.002). This correlation was the most significant in Seoul Metropolitan City, Incheon Metropolitan City, and Gyeonggi-do, and the least significant in Jeollanam-do and the southern region of Gyeongsangnam-do. For every 1% increase in the mean rate of lifetime diagnosis of allergic rhinitis, one of the variables in the disease contraction domain, the rate of experiencing depressive symptoms increased by 0.17%. Unlike myocardial infarction, the correlation between allergic rhinitis and the rate of experiencing depressive symptoms was the least significant in the capital area, and was the most significant in Gyeongsang Province. The weekly breakfast intake frequency of 1-2 times had a reduced correlation in the final model that considered the effects of disease contraction, and the social and physical environments in comparison to the model that only considered health behaviors. However, the rate of experiencing depressive symptoms was found to significantly increase with the rate of the weekly breakfast intake frequency of 1-2 times per week, indicating that populations with infrequent breakfast intakes are at higher risk for experiencing depressive symptoms (Table 3). The perceived stress level was the most correlated with the rate of experiencing depressive symptoms in Seoul Metropolitan City, Incheon Metropolitan City, and relatively less correlated in Gyeongsang Province. Among the social and physical environments, the public transit environment showed similar distributions of the correlation coefficient as the rate of lifetime diagnosis of allergic rhinitis; it was the least correlated with the rate of experiencing depressive symptoms in the capital area, and the most correlated in Gyeongsang Province (Figure 1). Comparison between the variables in the final model and those in the individual model of each domain showed differences in the correlation coefficients, but similar regional distributions between the variables, confirming the validity of the models. However, opposite results were obtained for the correlations between public transit environment and regional distribution, indicating that the influence of the former was not sufficiently explained in the analysis of the models of different domains.

**Figure 2.**
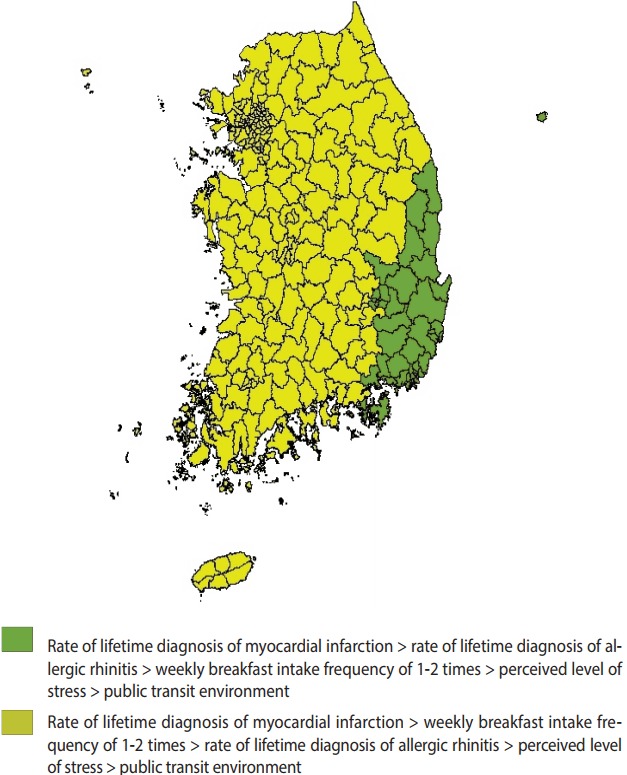
Classification based on the ranking of influence factors.

The five variables were associated with the rate of experiencing depressive symptoms to varying degrees, and the ranking of these variables by their correlational levels varied across regions. In all regions, the rate of lifetime diagnosis of myocardial infarction was the most correlated with the rate of experiencing depressive symptoms, while the public transit environment was the least correlated. The regions were divided into two groups according to the ranking of the variables by their level of correlation with the rate of experiencing depressive symptoms (Figure 2).

**Figure 3.**
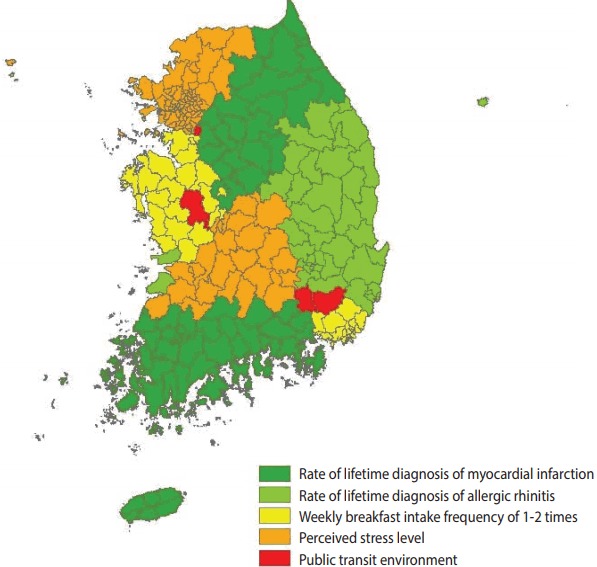
Classification based on the relative ranking of influence factors.

Rankings of the variables by the level of correlation with the rate of experiencing depressive symptoms were compared across the regions. The rate of lifetime diagnosis of myocardial infarction was ranked first in Gangwon-do, Chungcheongbuk-do, Jeollanam-do, and Gyeongsangnam-do. The rate of lifetime diagnosis of allergic rhinitis was ranked first in Gyeongsangbuk-do. The weekly breakfast intake frequency of 1-2 times was ranked first in Chungcheongnam-do and Ulsan Metropolitan City. The perceived stress level was ranked first in Seoul Metropolitan City, Incheon Metropolitan City, Gyeonggi-do, and Jeollabuk-do. The public transit environment was ranked first in five regions including Seoul Metropolitan City (Figure 3).

### Adequacy of the model

The GWR and ordinary least square (OLS) results of the three models for correlations between depressive symptoms and each domain, and the final model constructed based upon these models were compared. The R^2^-value, which describes the explanatory power of a model, was significantly high for all four models in GWR than OLS. However, the adjusted R^2^-value was slightly higher for the three models of the social and physical environments in OLS. AIC value, which measures the adequacy of a model, was lower in GWR than OLS for all models. Based on the overall results regarding the explanatory power and adequacy of the models, we decided that GWR analysis was more appropriate (Table 4).

Comparison of the models of different domains showed that model 4 or the final model had the highest explanatory power. Also model 4 had the lowest AIC value was therefore the most adequate. A Jarqure-Bera test was performed to determine whether OLS models satisfied the assumption of normality of the error distribution. The statistics of Jarqure-Bera for all four models were not statistically significant, meaning that the assumption was satisfied in all these models. The Koenker statistics, which tests the homoscedascity, were statistically significant for models 1 and 4, indicating that the correlations between the dependent variables and the explanatory variables were non-stationary, and exhibited spatial heterogeneity among the regions (Table 4).

## DISCUSSION

In this study, the extremal quotients of the rate of lifetime diagnosis of chronic disease, diabetes, cerebral infarction (stroke), and myocardial infarction were 1.78, 2.05, 4.40, and 5.60, respectively, and did not vary greatly among the areas. Conversely, the rate of experiencing depressive symptoms showed an extremal quotient value of 10.29 and huge regional disparities. The regional analysis of the rate of experiencing depressive feelings was thus meaningful.

Data of each administrative area were analyzed. However, there was a possibility of errors in which the effects of factors that were actually associated with depressive symptoms were reduced due to the arbitrary classification of administrative areas. Furthermore, to reflect the reality of the modern society in which people frequently change their residential, workplace, and academic environments due to the development of public transit, the influence of distances that could be travelled had to be considered. A GWR analysis was performed to minimize these errors and satisfy the assumption of normality of error distribution.

In the analysis of the models of different domains, no significant differences in the coefficient of determination, which measures the explanatory power of a model, and the AIC value, which assess the adequacy of a model, were found between the models of health behavior and disease contraction, and the final model. This means that health behaviors and disease contraction are closely associated with depressive symptoms, and the variables of these models reflected regional characteristics that were not included to a certain extent. However, the explanatory power and adequacy of the model of the social and physical environments were significantly lower than those of the final model, meaning that experience of depressive symptoms cannot be explained by environmental factors alone. While numerous studies have explained the associations between various health behaviors including smoking and drinking, and depression [25], most variables of smoking and drinking could not explain the rate of experiencing depressive symptoms in the present study. The perceived stress level was a factor that influenced the incidence of depressive symptoms as is reported in previous studies [29,30]. However, the results of this study regarding the correlation between depressive symptoms and exercise were contrary to those of a previous study [10]. In this study, as the standardized rate of walking increased, the rate of experiencing depressive symptoms increased; however, this correlation was not statistically significant, and could not be clearly explained. The weekly breakfast intake frequency of 1-2 times appears to explain a regular lifestyle rather than a nutritional status. A study has previously reported high incidence of depression in subjects with irregular breakfast intake [10]. An analysis of correlations between other diseases and depressive symptoms showed no significant correlation between chronic diseases such as diabetes and experience of depressive symptoms. This may be because while chronic diseases are considerably important, they are nationally well-managed today [31], and do not substantially interfere with daily activities. Myocardial infarction, which was shown to have a significant influence on experience of depressive symptoms, affects a vital organ of the body, and its recurrence can be lethal [32]. Management of myocardial infarction greatly restricts daily activities, and may be thought to be associated with psychological problems [33]. The rates of lifetime diagnosis of atopic dermatitis and allergic rhinitis were also significantly associated with experience of depressive symptoms. This is consistent with the results of a previous study in which depression levels and hostility were high among subjects with allergic diseases [34]. While atopic dermatitis has been a disease of interest in the modern society and has been actively managed, allergic rhinitis either causes serious impairments or symptoms commonly experienced by many people; hence, the seriousness of allergic rhinitis has been overlooked. However, approximately 80% of all patients with atopic dermatitis develop respiratory or allergic diseases such as asthma and rhinitis [35]. Therefore, the two diseases may be highly associated with one another in terms of their physiological mechanisms, and management of allergic rhinitis is necessary.

The social and physical environments were associated with experience of depressive symptoms to a certain degree. Regarding the public transit environment, as the level of satisfaction with the public transit increased, the rate of experiencing depressive symptoms decreased. Considering that urbanization and the development of public transit are positively correlated, the incidence of depressive symptoms can be estimated to be higher in non-urban areas. This interpretation is consistent with results of numerous studies that have assessed the gravity of depressive symptoms in rural areas [36,37].

In this study, rankings of the variables associated with depressive symptoms varied across regions. The rankings for the eastern region of Gyeongsang Province were different from those of other regions. In most regions, depressive symptoms were more correlated with disease-related variables than health behaviors, whereas the rate of lifetime diagnosis of myocardial infarction was the most correlated in the eastern region of Gyeongsang Province, followed by breakfast intake frequency. Ranking variables based on the significance of their correlation with a variable of interest is important for determining priorities and budget allocation in regional projects, and identifying regions with high levels of correlation is important for national projects.

However, because regions or populations were the unit of analysis in this epidemiological study, there is a possibility of ecological errors. Therefore, while the results of this study may be useful for the initial investigation process of targets of health services in regional interventional projects, more detailed analyses are necessary to use the results to select intervention targets that are actually effective.

Comparison of the explanatory power and adequacy of the models showed that OLS is more appropriate than GWR in an analysis that considers regional disparities and physical proximity. Health behaviors and disease contraction, whose close associations with depressive symptoms could be indirectly explained through comparison of the models of different domains and their explanatory power, had smaller explanatory power than the final model, but the difference was not statistically significant. Conversely, the explanatory power of the social and physical environments was significantly lower than the final model, demonstrating that depressive symptoms cannot be explained based on the social and physical environments alone. Moreover, the validity of the methodology used to establish the final model consisting of general health indicators was confirmed by constructing a model for each domain.

While the GWR analysis used in this study can produce results that reflect spatial heterogeneity through the use of geographical information, it puts a huge limitation during the process of building the models. By constructing a model for effective prevention and management projects for depressive symptoms through sufficient literature review and consideration of the possibility for intervention, basic research data that are more accurate and valid than those obtained from a conventional regression analysis may be obtained. Despite this limitation, the present study is meaningful in that it is an empirical study on depressive symptoms using a methodology that is not yet widely used in the field of public health, and that it considers the impact of regional variations. Effective regional health promotion programs may be planned based on the basic research data obtained in this study.

## Figures and Tables

**Table 1.** Descriptive statistics of indicators related to depression

Indicators	Mean	Min	Max	SD	Extremal quotient
Rate of experiencing depressive symptoms	5.07	1.05	10.8	2.13	10.29
Rate of exposure to secondhand smoke at home	11.21	4.30	22.00	2.99	5.12
Annual rate of drinking	73.53	53.25	82.75	5.93	1.55
Monthly rate of drinking	58.18	41.45	65.75	4.4	1.59
Age at drinking onset (yr)	21.23	20.05	22.65	0.49	1.13
Rate of walking	40.55	15.25	69.30	10.44	4.54
Rate of performing vigorous exercise	14.46	7.35	31.80	3.92	4.33
Rate of performing muscle strengthening exercise	18.28	6.15	33.15	3.96	5.39
Rate of performing moderate to high intensity physical activities	13.49	3.60	54.45	7.80	15.13
Level of fruit consumption	42.43	20.95	69.00	7.68	3.29
Weekly breakfast intake frequency of 1-2 times	5.94	1.85	9.50	1.57	5.14
Rate of consulting a psychologist due to stress	5.48	0.80	12.65	2.00	15.81
Perceived stress level	27.11	15.05	35.55	3.89	2.36
Rate of sleeping less than 6 hours per day	42.89	25.3	54.05	6.17	2.14
Rate of lifetime diagnosis of hypertension	14.89	10.95	19.50	1.66	1.78
Rate of lifetime diagnosis of diabetes	5.69	4.00	8.20	0.75	2.05
Rate of lifetime diagnosis of cerebral infarction (stroke)	1.08	0.50	2.20	0.29	4.40
Rate of lifetime diagnosis of myocardial infarction	0.79	0.25	1.40	0.23	5.60
Rate of lifetime diagnosis of atopic dermatitis	2.88	0.30	6.15	1.02	20.5
Rate of lifetime diagnosis of allergic rhinitis	12.88	1.85	21.25	3.7	11.49
Rate of lifetime diagnosis of asthma	2.10	0.80	3.80	0.59	4.75
Rate of lifetime diagnosis of angina	1.06	0.4	1.95	0.28	4.88
Rate of not receiving necessary medkal servkes	13.61	2.7	25.3	3.41	9.37
Contact with social networks (neighbors) less than once per month	33.31	0	68.7	17.61	0.00
Leisure environment	29.3	14.3	61.35	7.47	4.29
Religious environment	26.13	11.15	44.1	6.41	3.96
Social environment	56.77	40	77.85	7.44	1.95
Public transit environment	65.05	29	93.1	14.45	3.21
Supportive environment	51.87	15.4	95.65	22.9	6.21
Trust environment	66.41	39.65	97.9	11.49	2.47
Safety environment	75.35	48.5	95.05	9.56	1.96
Healthcare environment	62.82	23.35	90.4	13.33	3.87
Natural environment	80.3	36.6	97.8	11.51	2.67

Min, minimum; Max, maximum; SD, standard deviation.

**Table 2.** Correlation between indicators and depression and spatial autocorrelation

Indicators	Correlation		Spatial autocorrelation		
	Coefficient	p-value	Moran's I	Z-score	p-value
Rate of experiencing depressive symptoms	-	-	0.34	27.85	<0.001
Rate of lifetime diagnosis of depression	-	-	0.06	5.47	<0.001
Rate of exposure to secondhand smoke at home	0.24	0.0001	0.03	3.18	0.002
Rate of exposure to secondhand smoke in public	0.41	<0.001	0.20	16.74	<0.001
Annual level of drinking	0.51	<0.001	0.33	26.96	<0.001
Monthly level of drinking	0.46	<0.001	0.32	26.09	<0.001
Age at drinking onset (years)	-0.49	<0.001	0.36	29.46	<0.001
Rate of walking	0.20	0.001	0.37	30.25	<0.001
Rate of performing rigorous physical activities	-0.29	<0.001	0.06	5.39	<0.001
Rate of performing muscle strengthening exercise	0.55	<0.001	0.43	34.63	<0.001
Rate of performing moderate to high intensity physical activities	-0.35	<0.001	0.28	22.63	<0.001
Level of fruit consumption	0.33	<0.001	0.28	23.05	<0.001
Weekly breakfast intake frequency of 1-2 times	0.59	<0.001	0.37	30.09	<0.001
Rate of consulting a psychologist due to stress	0.45	<0.001	0.10	8.32	<0.001
Perceived stress level	0.57	<0.001	0.32	25.96	<0.001
Rate of sleeping less than 6 hours per day	0.58	<0.001	0.59	47.3	<0.001
Rate of lifetime diagnosis of hypertension	-0.35	<0.001	0.59	47.30	<0.001
Rate of lifetime diagnosis of diabetes	0.21	0.002	0.06	4.98	<0.001
Rate of lifetime diagnosis of cerebral infarction (stroke)	0.20	0.001	0.03	3.04	0.002
Rate of lifetime diagnosis of myocardial infarction	0.37	<0.001	0.04	3.83	<0.001
Rate of lifetime diagnosis of atopic dermatitis	0.59	<0.001	0.54	43.12	<0.001
Rate of lifetime diagnosis of allergic rhinitis	0.65	<0.001	0.42	34.14	<0.001
Rate of lifetime diagnosis of asthma	0.41	<0.001	0.12	10.26	<0.001
Rate of lifetime diagnosis of angina	0.23	<0.001	0.07	5.81	<0.001
Rate of not receiving necessary medical services	0.40	<0.001	0.00	0.38	0.70
Contacting social networks (neighbors) less than once per month	-0.47	<0.001	0.51	40.66	<0.001
Leisure environment	0.41	<0.001	0.34	27.47	<0.001
Religious environment	0.24	<0.001	0.44	27.47	<0.001
Social environment	-0.47	<0.001	0.39	31.12	<0.001
Public transit environment	0.28	<0.001	0.41	33.38	<0.001
Supportive environment	-0.47	<0.001	0.49	39.61	<0.001
Trust environment	-0.55	<0.001	0.5	40.42	<0.001
Safety environment	-0.47	<0.001	0.31	25.42	<0.001
Healthcare environment	0.14	0.002	0.22	18.16	<0.001
Natural environment	-0.40	<0.001	0.33	26.4	<0.001

**Table 3.** Association between community characteristics and depression

Model	GWR (n=251)					OLS (n=251)		
	Mean	Min		Max	SD	B		p-value
Constant	-1.88	-3.31		-0.42	1.04	-1.98		0.001	
Rate of lifetime diagnosis of myocardial infarction	1.21	0.48		1.59	0.34	1.12		0.002	
Rate of lifetime diagnosis of allergic rhinitis	0.17	0.13		0.23	0.03	0.18		<0.001	
Weekly breakfast intake frequency of 1-2 times	0.26	0.17		0.35	0.05	0.24		<0.001
Perceived stress level	0.1	0.07		0.13	0.02	0.10		<0.001	
Public transit environment	-0.01	-0.02		0.00	0.01	-0.01		0.006	
R-squared			0.58				0.54		
Adjusted R-squared			0.55				0.53		
AIC			808.71				815.42		
Normality (Jarque-Bera)			7.06^*^						
Homoscedasticity (Koenker-Bassett)			9.25						

GWR, geographically weighted regression; OLS, ordinary least square; Min. minimum; Max, maximum; SD, standard deviation; AIC, Akaike’s information criterion.

* p<0.05

**Table 4.** Comparative analysis of models

Model	Comparative analysis	Model 1^1^	Model 2^2^	Model 3^3^	Model 4^4^
GWR	R-squared	0.53	0.52	0.35	0.58
	Adjusted R-squared	0.5	0.5	0.33	0.55
	AIC	839.36	833.14	905.07	808.71
OLS	R-squared	0.48	0.5	0.31	0.54
	Adjusted R-squared	0.47	0.49	0.35	0.53
	AIC	847.83	836.32	913.91	815.42
	Koenker statistics	19.08^*^	5.74	5.58	7.06^*^
	Jarqure-Bera statistics	4.39	5.23	6.54	9.25

GWR, geographically weighted regression; OLS, ordinary least square; AIC, Akaike’s information criterion.

1 A model consisting of health behavior indicators.

2 A model consisting of disease indicators.

3 A model consisting of social-physical environment indicators.

4 Final model.

* GWR, geographically weighted regression; OLS, ordinary least square; AIC, Akaike’s information criterion.
