# Supplementary material for: Analysis of the relationship between community characteristics and depression using geographically weighted regression
Source: Epidemiol Health. 2017 Jun 21;39:e2017025. doi: 10.4178/epih.e2017025 (PMC5668667; doi:10.4178/epih.e2017025)
Supplement: Supplementary file 1 [file epih-39-e2017025-supplementary.pdf]

Volume: 39, Article ID: e2017025

https://doi.org/10.4178/epih.e2017025

지리가중회귀모형을 이용한 지역사회 특성과 우울증의 관계 분석

Analysis of the Relationship between Community Characteristics and Depression Using Geographically Weighted Regression

최형윤1, 김호2,3

1 질병관리본부 질병예방센터 만성질환관리과,

2 서울대학교 보건대학원

3 서울대학교 아시아에너지환경지속가능발전연구소(AIEES)

교신저자: 김호, 서울특별시 관악구 관악로1 서울대학교 보건대학원

Tel: 02-880-2711/E-mail: hokim@snu.ac.kr

Volume: 39, Article ID: e2017025

https://doi.org/10.4178/epih.e2017025

Abstract

Objectives: 온 국민이 누리는 건강 세상을 위해 건강형평성의 확보는 시급한 현안이며 실제로 많은 건강결정요인은 지역별 차이를 보이고 있다. 특별히 정신질환 중 가장 흔한 우울증의 경우, 집단의 특성 간 발생 현황에 차이를 보이고 있어 지역별 접근을 통한 연구가 요구됨에 따라 본 연구에서는 우울증의 기저 감정인 우울감 경험의 지역적 변이를 분석하여 지역별 중재가 필요한 건강결정요소를 파악하고자 하였다.

Methods: 지역사회건강조사 2011년, 2013년 자료 중 우울증과 관련 있는 지표를 분석대상에 포함시켰다. 이의 변수들에 대해 Moran의 I 지수를 산출하여 공간적 자기 상관성을 확인 후 공간적 지리가중회귀분석의 타당성을 판단하여 ArcGIS 10.1을 사용한 지리가중회귀분석을 시행하였다. 최종 모형은 건강행태, 이환, 사회·물리적환경 모형을 별도 구축한 후 유의한 변수 조합을 통해 구성하였다.

Results: 건강행태 영역에서는 모든 지역에서 아침식사 빈도 주 1~2회 변수가 가장 관련성이 높은 것으로 나타났으며 그 다음으로는 지역에 따라 간접흡연 노출률 또는 스트레스 인지율인 것으로 나타났다. 이환 영역에서 가장 관련이 큰 것은 심근경색증 평생의사진단 경험률이었으며 사회. 물리적 환경 영역에서는 지역사회 신뢰 환경이 가장 연관성이 높아 신뢰가 낮을수록 우울감 경험이 높아진다는 결과가 도출되었다. 영역별 영향이 높은 변수들로 최종 모형을 구성하여 분석한 결과, 우울감 경험과 관련이 높은 순위에 따라 2개 그룹으로 분류할 수 있었다.

Conclusions: 본 연구를 통해 지역단위보건관련지표는 지역의 우울감 경험 발생과 유의미한 연관성이 있으며 연관성 우선순위에도 지역별 차이가 존재함을 알 수 있었다. 지역적 특성에 따른 우선순위를 제시하였음에 본 연구의 의의가 있으며 공중 보건 영역의 다른

사례에 본 연구방법론 및 연구결과 제시 방안을 적용함에 따라 지역의 건강수준향상 프로그램 개발에 유용한 기초자료의 제공을 기대할 수 있다.

keywords : Depression, Depressive disorder, Spatial regression, Spatial analysis , Health status

서론

온 국민이 누리는 건강 세상을 위해 건강형평성의 확보는 시급한 현안이다. 건강형평성 확보란 건강불평등의 해소를 의미하며 건강불평등이란 인구집단 간 건강상태가 불균등하게 분포되어 있는 것을 뜻한다[1]. 건강불평등이 흔하게 나타나는 형태 중 하나로 지역 간 차이를 꼽을 수 있으며[2] 실제로 많은 건강관련지표 및 건강결정요인이 지역별 차이를 보이고 있다[3,4]. 그럼에도 불구하고 건강수준의 지역별 요인 분석에 관한 연구 사례는 드문 실정이다. 최근 국내에서 이를 고려한 연구결과가 제시되고 있으나 거의 대부분 만성질환에 한해 다루고 있다[5-9]. 만성질환의 경우 많은 사람들이 관심을 가지고 국가차원에서도 꾸준히 관리하는 단계에 이르렀으나 정신질환은 개인 수준에서뿐만 아니라 사회적 문제로 대두되고 있음에도 불구하고 그 심각성에 대한 인지 및 관리율이 낮은 실정이다[10]. 특별히 정신질환 중 가장 흔한 우울증의 경우, 집단의 특성간 발생 현황에 차이를 보이고 있어[11,12] 지역 간 차이도 있을 것으로 사료됨에 따라 지역별 접근을 통한 연구가 요구된다.

우울증은 만성질환보다 개인의 사회적, 신체적 안녕을 더욱 손상시키는 질환으로 밝혀진 바 있으며[13], 가족관계, 사회생활 등에 부정적인 영향을 미쳐 전반적인 삶의 질을 떨어뜨리고 장애를 유발하여 유병자 중 10-15%는 결국 자살에 이르게 할 정도로 심각한

Volume: 39, Article ID: e2017025

https://doi.org/10.4178/epih.e2017025

질환이다[14]. 국제협력개발기구(OECD) 통계에 따르면, 2013년 기준, OECD 주요 국가들의 연령표준화 자살률 평균은 인구 100,000명 당 12.1명인데, 한국의 경우 27.3명으로 평균 자살률의 두 배가 넘는 수치를 기록하였다. 한편, 국제질병부담(Global Burden of Diseases, GBD) 연구 결과 291개 질병의 장애보정손실연수(Disability adjusted life years, DALY) 순위는 1990년을 기준으로 우울증(Major depressive disorder)이 15위였으며[15] 2010년 11위로 높아졌다. 특히 장애보정 손실연수(DALY)는 조기 사망에 따른 손실연수(Years of Life Lost, YLL)와 장애생활연수(Years lived with Disability, YLD)의 합으로 산출되는데, 이 때 장애생활연수의 순위는 1990년 3위, 2013년 2위로 높아졌으며, 이의 결과를 통해 우울증이 직접적으로 사망에 미치는 영향은 치명적이지는 않지만 건강한 삶의 측면에서는 매우 부정적인 영향을 미치고 있다는 해석이 가능하다. 이처럼 우울증은 자살의 주요인으로 작용하는 것 이외에도 여러 부정적 결과를 초래하기 때문에 국민의 삶의 질 향상뿐만 아니라 장기전인 국가 발전의 차원에서도 관리가 필요하다.

우울증은 성별, 연령 등 개인의 생물학적 취약성[16,17],], 사회활동 참여[20] 등의 사회적 자본[18,19], 지역사회 신뢰환경[21,22], 만성질환여부[23,24], 흡연[25], 음주, 운동, 수면 시간 등 다양한 건강행태[10]와 관련이 있는 것으로 알려져 있다. 우울증에 영향을 미치는 이의 요인들은 대부분 지역별 상이한 분포를 나타냈으며[26] 이에 따라 우울증 발생 양상에 지역 간 차이가 발생할 것으로 예상된다. 이러한 요인들은 중재가 가능한 요인으로서 이를 파악하는 것은 건강증진 측면에서 의미가 있다.

이의 필요성에 따라 본 연구에서는 우울증의 지역적 변이요인을 분석하여 지역별 중재가 필요한 건강결정요인을 파악하고, 나아가 우울증 사례뿐만 아니라 보건영역에서 지역적 차이를 고려하여야 하는 다른 건강수준 사례에도 적용이 가능하도록 연구방법론 및

Volume: 39, Article ID: e2017025

https://doi.org/10.4178/epih.e2017025

연구결과제시 방안을 제안하고자 한다.

연구방법

연구자료

본 연구는 지역사회특성과 우울증의 관계를 규명하기 위하여 시·군·구 단위의 지표를 제공하는 지역사회건강조사 자료를 사용하였으며 시의 확보를 위해 최신 가용 자료인 2013년 자료를 기본으로 하였다. 그러나 주제의 특성상 여러 건강행태 관련 변수와 종속변수 간 역인과 발생 가능성이 예상됨에 따라 종속변수는 2013년, 독립변수는 2013년과 과거 연도의 평균값을 사용하였다. 이 때 변수 중 2년 주기 조사항목이 존재함에 따라 과거 연도는 2011년 자료를 사용하였다. 매년 독립적인 표본으로 구성된 대표적인 단면조사자료(repeated cross-sectional survey data)라는 점을 감안할 때 표본추출 및 설문조사 과정에서의 오류 발생 가능성이 있어[7] 이의 보완이 필요하였으며 지역보건사업은 장기적 관점에서의 중재이기 때문에 다년자료의 활용이 단년자료 사용보다 더 타당한 것으로 판단하였다.

지역사회건강조사 자료의 우울증 관련된 지표는「우울증 평생의사진단 경험률」과「우울감 경험률」이 있다.「우울증 평생의사진단 경험률」은 의사의 진단에 따른 값으로 질병유무가 검증된 사례에 대한 측정치이기는 하나, 병·의원에 방문한 경우에만 해당되어 정신과적 질환의 특성을 고려할 때 타 실제 질환을 앓고 있는 경우 보다 과소 측정될 가능성이 크다는 한계가 있으며 타 질환의 의사진단 경험률보다 그 정도가 크며 질환의 유무 이외 혼란을 야기하는 요인 또한 많을 것으로 판단하였다. 반면「우울감 경험률」의 경우, 우울증의 기저 감정에 대한 지표로서 정확히 의사로부터 진단받은 경우는 아니지

Volume: 39, Article ID: e2017025

https://doi.org/10.4178/epih.e2017025

만 “최근 1년 동안 연속적으로 2주 이상 일상생활에 지장이 있을 정도로 슬프거나 절망감 등을 느낀 적이 있습니까?”에 대한 응답값으로 실제 우울증 진단 기준을 질문에 일부 반영하고 있기에 이를 우울증을 설명하는 종속변수로 선택하였다. 미국 정신의학협회의 정신질환 분류기준(DSM-IV)에 따르면 하루 종일 거의 매일 우울한 기분의 지속, 모든 즐거움과 흥미의 현저한 상실, 식욕감퇴로 인한 현저한 체중감소 및 증가, 매일 계속되는 불면증 또는 수면과다, 정신운동의 변화, 피로감 및 에너지 상실감, 무가치감 혹은 부적절한 죄책감, 사고력이나 집중력 및 판단력의 저하, 죽음에 대한 반복적인 생각과 자살 계획 및 시도 증상 중 5가지 이상이 2주 동안 지속되고 이전의 기능수준과 다른 변화가 나타날 때 우울증으로 분류한다[27].

분석에 포함될 독립변수는 지역사회건강조사 자료 중 문헌고찰을 통해 우울증과 관련이 있다고 알려진 변수를 1차로 선정 후 종속변수와 상관성이 높은 지표를 선별하였다.

최종 가용 변수는 종속변수를 포함하여 총 50개였으며 시·군·구 지역의 수는 2011년과 2013년 기간 중 행정구역 통폐합으로 인해 정확하게 일치하지 않는 경우 2013년 기준의 행정구역 기준으로 지역의 정의를 조정하여 총 251개 지역을 연구 대상으로 하였다.

통계적 분석

모든 지표는 정확한 지역 간 비교가 가능하도록 2005년 추계인구를 사용하여 연령을 10세간격인 19-29세, 30-39세, 40-49세, 50-59세, 60-69세, 70세 이상으로 구분하여 연령별 성별 직접표준화를 하였다.

문헌고찰을 통해 선별한 우울증과 관련 있을 것으로 판단된 지표와 종속변수인 「우울감 경험률」의 연관성 확인을 위해 상관분석을 하였으며 상관계수가 5% 유의수준에서 통

Volume: 39, Article ID: e2017025

https://doi.org/10.4178/epih.e2017025

계적으로 유의하게 절댓값 0.2 이상인 변수를 공간분석 대상으로 하였다. 통계적 유의성이 확보되지 않은 변수 중 중재의 가능성 및 보건학적 의미가 큰 변수는 분석에 포함하였다.

본 연구 자료는 시·군·구 행정구역 단위로 수집된 자료로서 실제 영향요인이 행정구역 분류로 인해 차단될 수 있음에 따라 물리적 인접성 반영이 필요하며 이를 위해 기본적으로 변량의 크기가 유사한 지역끼리 서로 이웃하고 있는지에 대한 확인이 필요하여 등간척도로 측정된 공간패턴을 전역적 Moran의 I 지수를 통해 파악하여 지리가중회귀분석의 타당 여부를 판단하였다. Moran의 I 지수를 통해 공간적 자기 상관을 확인하였음에 따라 지리가중회귀분석을 시행하였다. 이때 모형의 적합도는 대역폭의 설정에 의해 영향을 받게 되는데 표본의 분포 패턴이 규칙적일 경우 고정된 커널(fixed kernel)을 사용하며 분포가 불규칙적일 경우 가변적 커널(adaptive kernel)을 사용하여 모형의 적합도를 높이게 된다[28]. 본 연구에서는 지역간 거리가 동일하지 않으므로 표본간 거리가 가까울 때 대역폭을 작게, 비교적 거리가 멀 때에는 대역폭을 넓게 설정하기 위해 가변적 커널방식으로 대역폭을 설정하고 일반적 사용이 용이한 AIC를 채택하여 모형의 적합도를 판정하였다.

지리가중회귀모형은 3개의 영역으로 구분하여 영역별 분석 시행 후 영역별 분석 결과를 통해 유의하게 영향이 있는 변수들로 최종연구모형을 구축하였다. 이는 엄격한 다중공선성 기준 적용에 따라 모형에 우선적으로 포함되는 변수의 결정에 추가할 수 있는 변수가 제한되므로 영역별 편향이 발생을 보완하기 위한 방볍이다. 분석에는 ArcGIS 10.1이 사용되었다.

지리가중 회귀분석을 산출된 우울증에 영향을 미치는 변수들 중 타 지역에 비해 영향의 정도가 가장 높은 변수를 파악하기 위하여 지역별 회귀계수들을 Z 점수로 변환하여 변수 간 비교를 하였다. Z 점수는 표준점수의 하나로 평균으로부터의 편차 점수를 그 분포의 표준편차로 나누어 상대적 의미에서의 비교하였으며 변수 별 지역의 Z 점수를 산출하여 순위로 변환한 후 지역 내 여러 변수 중 순위가 가장 높은 변수에 대해 파악하였다.

연구결과

우울증 관련요소 지역별 격차 현황

지역별 격차를 확인할 수 있는 극단값비의 경우, 중등도 신체활동 실천율, 스트레스로 인한정신상담률, 아토피피부염 및 알레르기성 비염 평상의사진단 경험률이 높게 나타났다[table 1].

지역별 군집 유형 공간적 연관성

우울감 경험률과 관련이 있는 변수를 파악하기 위한 상관분석 결과 5% 유의수준에서 통계적으로 유의하게 연관성을 보인 변수 총 33개였다. 이 중 가장 연관성이 큰 것으로 나타난 변수는 상관계수 0.65인 알레르기성 비염 평생 의사진단 경험률이었다[table 2].

변수들의 공간적 연관성을 확인하기 위한 공간적 자기상관성 분석 결과, 상관분석을 통해 선별한 대부분의 변수가 5% 유의수준에서 통계적으로 유의하게 양의 자기상관 관계를 보여 지리 가중 회귀분석에 적합하다는 판단을 하였다. 특히 종속변수인 우울감 경

Volume: 39, Article ID: e2017025

https://doi.org/10.4178/epih.e2017025

험률의 경우 Moran의 I 지수가 0.34로 군집성이 나타났다[table 2].

지역사회 특성과 우울감

지역사회의 여러 보건학적 특성 중 우울감 경험과 연관이 깊은 요소를 다각적으로 파악하기

위해 각 영역별 관련성이 높은 변수로 아래 식과 같이 최종 모형을 확정하였다

$$\begin{aligned} Y_{\text{우울감경험률}} = B_0 &+ B_1(\text{심근경색증 평생 의사진단경험률}) \\ &+ B_2(\text{알레르기성 비염 평생 의사진단경험률}) \\ &+ B_3(\text{아침식사빈도주1}-2\text{회}) \\ &+ B_4(\text{스트레스 인지율}) \\ &+ B_5(\text{지역사회 대중교통환경}) + \varepsilon \end{aligned}$$

모형에 포함된 5개의 변수는 모두 통계적으로 유의한 연관성이 있었다. 변수 중 가장 큰 연관성을 보이는 것은 심근경색증 평생 의사진단 경험률이었으며 유의확률이 0.0021로 통계적으로 유의하게 심근경색증 평생 의사진단 경험률이 1% 증가한 지역은 우울감 경험률이 1.21% 증가하는 것으로 나타났다. 이는 서울특별시, 인천광역시, 경기도 지역에서 가장 크게 나타났으며 전라남도, 경상남도인 남부 지역에서 연관성이 가장 낮은 것으로 나타났다. 이환 영역 내의 다른 질환인 알레르기성 비염 평생 의사진단 경험률의 경우, 평균적으로 1% 증가함에 따라 우울감 경험률은 0.17% 증가하였으며 심근경색증의 지역 분포와 달리 수도권 지역에서 연관성이 가장 낮았고 경상도 지역에서 가장 크게 나타났다. 건강행태 영역에서 우울감 경험률과 가장 연관성이 높은 것으로 확인되었던 아침식사 빈도 주 1~2회 변수의 경우, 이환과 사회.물리적 환경의 영향을 고려함에 따라 건강행태만을 고려하였던 모형에서의 결과보다 관련성의 정도가 줄었으나 통계적으로 유의하게 본 변수의 증가에 따라 우울감 경험률도 증가하는 결과를 보여 아침식사를 잘 하지 않는 집단일수록 우울감을 경험할 확률이 높아짐을 확인할 수 있었다[table 3]. 스트

레스 인지율의 경우 서울특별시, 인천광역시 지역에서 연관성이 가장 크게 나타났으며 경상도 지역에서 비교적 낮게 나타났다. 사회.물리적 환경 중 대중교통 환경은 알레르기성 비염 평생의사 진단률과 우울감 경험률의 연관성과 유사한 지역 분포로 수도권 지역에서 가장 낮았고 경상도 지역에서 영향이 가장 크게 나타났다[Figure 1]. 본 모형에 포함된 변수들의 분석 결과를 각 영역별 모형 분석 시 나타난 결과 비교하여 보면 회귀계수의 차이는 있으나 유사한 지역분포를 분포를 보여 모형의 구성이 타당함을 설명할 수 있으나 지역사회 대중교통 환경의 경우 영역별 모형 분석의 결과와 회귀계수의 부호 및 지역별 분포가 반대로 나타나 영역별 모형 분석 시 이의 영향에 대해 잘 설명하지 못했음을 알 수 있었다.

5개의 변수가 각 각 우울감 경험률에과 관련의 정도에는 차이가 있었으며 관련성의 순위가 지역별 상이함을 보였다. 전 지역에서 동일하게 우울감 경험률과 가장 관련이 높은 변수는 심근경색증 평생 의사진단 경험률, 가장 미미한 관련성을 보이는 변수는 지역사회 대중교통 환경이었다. 연관 정도의 우선순위에 따라 두 개의 그룹으로 분류되었다 [Figure 2].

다른 지역과 비교하여 상대적으로 우울감 경험률과 관련의 정도가 큰 지표의 순위를 분석한 결과, 심근경색 평생 의사진단 경험률이 1위인 지역은 강원도, 충청북도, 전라남도, 경상남도로 나타났으며 알레르기성 비염 평생의사 진단율이 1위인 지역은 경상북도, 아침식사빈도 주 1~2회가 1위인 지역은 충청남도, 울산광역시, 스트레스 인지율이 1위인 지역은 서울특별시, 인천광역시, 경기도, 전라북도로 나타났으며 지역사회 대중교통 환경이 1위인 지역은 서울특별시 1개 구를 포함하여 5개 지역으로 나타났다[Figure 3].

Volume: 39, Article ID: e2017025

https://doi.org/10.4178/epih.e2017025

모형의 적합성

우울감과 각 영역별 관련성 대한 3개의 모형과 이를 통해 구축한 최종모형에 대하여 GWR과 OLS를 비교 분석하였다. 모형의 설명력을 의미하는 R^2 는 4개의 모형에서 OLS보다 GWR의 결과에서 미미하지만 높게 나타났으나 adjusted R^2의 경우, 사회·물리적 환경에 대한 모형 3의 경우 OLS가 조금 더 설명력이 높은 것으로 나타났다. 한편 모형의 적합성을 나타내는 AIC는모든 모형에서 GWR의 값이 OLS보다 낮게 나타났다. 모형의 설명력이나 적합성의 종합적 확인을 통해 GWR을 통한 분석이 더 적합함을 확인하였다 [Table 4].

영역별 모형의 비교결과 모형의 설명력이 가장 좋은 모형은 모형4에 해당하는 최종모형이었으며 모형의 적합성 측면에서도 모형 4가 AIC값이 가장 낮아 가장 적합한 것으로 나타났다. OLS 모형이 가정을 준수하는 가를 진단하기 위해 오차의 정규성을 판정하는 Jarqure-Bera 통계량은 4개의 모형 모두 유의하지 않아 오차의 정규성 가정을 준수하고 있음을 확인하였다. 또한 등분산성 가정 준수를 확인하는 Koenker 통계량은 모형 1과 모형 4에서 유의하게 나타나 종속변수와 설명변수 간 관계들이 비고정성(non-stationary)이 있어 지역에 따라 관계가 달라지는 공간적 이질성을 갖고 있음을 확인하였다[Table 4]..

고찰

본 연구에서 만성질환의 의사진단 경험률의 극단값비를 살펴보면 고혈압은 1.78, 당뇨병의 경우 2.05, 뇌졸중(중풍)은 4.40, 심근경색증 5.60으로 지역별로 차이가 크지 않은데 반해 종속변수로 선정한 우울감 경험률은 극단값비가 10.29로 지역별로 큰 차이가 존재

Volume: 39, Article ID: e2017025

https://doi.org/10.4178/epih.e2017025

함을 알 수 있었다. 이로써 우울감 경험률에 대한 지역 단위 분석이 의미 있음을 확인할 수 있었다.

이의 필요에 따라 지역별 자료가 필요하였으나 행정구역 단위로 수집된 지역별 자료는 임의의 구분에 따라 실제 영향이 차단되는 오류 발생 가능성이 있다. 또한 지역별 분석 시 교통수단의 발달 등으로 주거지와 직장 및 학업 환경이 일치하지 않아 이동이 잦은 현대 사회의 현황을 최대한 반영하기 위하여 이동 가능한 거리의 영향에 대한 고려가 필요하였다. 이러한 발생 가능한 오류의 최소화와 발생 가능한 가정을 포괄하기 위해 지리 가중회귀분석을 시행하였다.

영역별 모형의 분석 결과, 건강행태 영역 모형과 이환 영역 모형은 최종 모형과 비교하였을 때 모형의 설명력을 나타내는 설명계수 및 적합성을 진단하는 AIC의 차이가 크게 나타나지 않았다. 이는 건강행태와 이환은 밀접한 연관이 있는 요소들로 해당 모형에 포함되지 않은 지역의 특성을 어느 정도 반영하는 변수들로 구성되었다고 생각할 수 있다. 그러나 사회.물리적 환경 영역 모형은 모형의 설명력이나 적합성이 최종 모형에 비해 현저하게 낮았고 이는 우울감 경험을 환경적인 요인으로만 설명할 수 없다는 해석을 가능하게 하였다. 건강행태와 우울감 경험의 관계 분석에 있어서 기존의 수많은 연구들이 우울증과 흡연 및 음주의 연관성에 대해 설명하고 있으나[25] 본 분석에서는 흡연 및 음주와 관련한 대부분의 변수가 우울감 경험을 설명하지 못하였다. 스트레스 인지율의 경우 스트레스가 우울감 발생에 영향을 미치는 요인임을 밝힌 기존의 연구들과 결과가 일치하였다[29,30]. 그러나 우울감 경험과 운동의 연관성을 밝힌 연구들[10]과 상반되는 결과를 보인 걷기실천율은 본 지표의 표준화율이 증가하면 우울감 경험도 증가하는 것으로 나타났는데 통계적으로 유의하지 않아 명확한 설명이 되지 않았다. 아침식사 빈도 주

Volume: 39, Article ID: e2017025

https://doi.org/10.4178/epih.e2017025

1~2회 변수는 영양 상태에 대한 해석보다는 규칙적인 생활을 설명하는 지표로 해석된다. 실제로 기존 연구에서 아침식사를 불규칙적으로 하는 군에서 우울증 유병률이 높음을 밝혀낸 바 있다[10]. 다른 질병과 우울감 경험의 관계 분석 결과 고혈압 및 당뇨병과 같은 만성질환은 우울감 경험과의 연관성이 크게 없는 것으로 나타났는데 이는 중요한 질환이기는 하지만 만성질환이 오늘날 국가 수준에서도 잘 관리되어[31] 일상생활에 불편함을 크게 주지 않는 질병이기 때문으로 생각된다. 본 분석에서 우울감 경험에 미치는 영향이 상당히 크게 나타난 심근경색증의 경우 생명과 직결된 부위의 장애 경험으로 재발 시 위험 가능성이 높은 것으로 알려진 바[32], 관리를 위해 일상생활에서의 제한이 많은 질병이기 때문에 정신적인 문제로의 연결을 생각할 수 있다[33]. 아토피 피부염과 알레르기성 비염 평생 의사진단 경험률 또한 우울감 경험과 관련성이 큰 것으로 나타났다. 이는 알레르기성 질환군에서 우울과 적대감이 많다는 기존의 연구결과와 일치한다[34]. 아토피피부염은 현대인에게 관심과 관리의 대상이 되었으나 알레르기성 비염의 경우 큰 장애를 초래하거나 많은 사람들이 겪어 본 증상이 나타나는 질병으로 간과하게 되는 질병 중 하나이다. 그러나 아토피 피부염 환자의 약 80% 정도는 후에 천식이나 비염 등의 호흡기 질환이나 알레르기 질환을 보인다는 점[35]과 같이 이 두 질병은 생리학적 기전에 있어서 연관성이 높은 질환으로 알레르기성 비염도 관리가 필요함을 시사한다. 한편, 사회. 물리적 환경은 정도가 크지 않지만 우울감 경험에 영향을 미치는 부분이 있는 것으로 나타났다. 지역사회 대중교통 환경의 경우 대중교통 환경에 만족하는 사람의 분율이 높을수록 우울감 경험이 감소한다는 결과를 보였는데 일반적으로 도시화와 대중교통의 발달에 양의 상관성이 있다는 사실을 미루어 볼 때 도시화가 되지 않은 곳에서 우울감 경험이 더 많이 발생한다고 해석할 수 있으며 이는 농촌 지역 우울감 경험의 심각성을 입증하는 많은 연구 결과와 맥락을 같이 한다[36,37].

Volume: 39, Article ID: e2017025

https://doi.org/10.4178/epih.e2017025

또한 본 연구에서 우울감 경험과 관련이 높은 순위는 지역별 차이가 있었는데 경상도 동부 지역이 다른 지역과 분포를 달리했다. 대부분의 지역에서 건강행태보다는 질병에 기인한 연관성이 크게 나타난 반면, 경상도 동부 지역에서는 심근경색증 평생 의사진단 경험률 다음으로 아침식사 빈도가 관련 높은 것으로 나타났다. 지역단위 사업의 우선순위 결정에는 지역에서의 연관성 정도의 우선순위가 중요하며 국가단위 사업의 우선순위 결정에는 관련의 정도가 특히 큰 지역을 파악하는 것이 중요하다. 이는 예산 배분의 근거가 될 수 있기 때문이다.

그러나 본 역학연구는 분석의 단위가 지역, 즉 집단이므로 생태학적 오류가 발생할 수 있다는 점에서 지역사회 중재사업을 위한 활용 시 보건사업대상의 1차 탐색과정에서는 유용할 것으로 사료되나 실제 효과적인 중재대상을 선정하기 위해서 활용하기 위해서는 더욱 심도 있는 분석이 필요할 것이다.

모형의 설명력과 적합성 비교 결과, 지역별 차이 및 거리의 인접성에 대한 영향을 고려한 분석 시 OLS보다 GWR이 적합하다는 결론을 얻었다. 영역별 모형의 설명력 비교를 통해 서로 밀접한 관계로 간접적 설명이 가능한 건강행태와 이환의 경우 최종 모형에 비해 설명력이 낮기는 하지만 큰 차이가 없는 반면 사회·물리적 환경 영역 모형의 경우 타 모형에 비해 설명력이 현저하게 떨어지는 것으로 보아 사회·물리적 환경으로만 우울감 경험을 설명할 수 없음을 확인하였다. 또한 이를 통해 영역별 구축을 통해 전반적인 보건지표로 구성한 최종모형을 구축한 방법론의 타당함을 확인하였다.

Volume: 39, Article ID: e2017025

https://doi.org/10.4178/epih.e2017025

본 연구에서 사용한 지리가중회귀 분석 방법은 지리 정보 활용을 통해 공간적인 이질성을 반영한 결과를 얻을 수 있는 반면, 모형 구축 시 큰 제한이 있었다. 이에 따라 충분한 문헌 고찰 및 중재 가능성 고려를 통해 효과적인 우울감 경험의 예방 및 관리 사업을 위한 모형을 구축한다면 일반회귀분석보다 더 정확하고 타당한 기초정보 생성할 수 있을 것으로 생각된다. 이러한 제한점에도 불구하고 본 연구는 우울감 경험 사례의 실증연구를 통해 지역별 차이 및 인접 지역으로부터의 영향을 고려한 아직까지 공중보건 분야에서 크게 활용되지 못하고 있는 분석 방법 및 결과도출 방안을 제시하였다는 측면에서 의의가 크다. 이를 통해 지역기반의 효과적인 건강증진 사업 기획을 기대할 수 있다.

참 고 문 헌

[1] Yoon TH. Regional health inequalities in Korea: the status and policy tasks. J Crit Soc Welf 2010;30:49-77.

[2] Kim HY. Community inequalities in health: the contextual effect of social capita. Korean J Sociol 2010;44:59-92 (Korean).

[3] Ko SJ. Factors of health inequalities by residential area differences. Natl Assoc Korean Local Gov Stud 2010;12:169-195 (Korean).

[4] Kim SY, Yoon KC. An analysis of the regional differences of health inequality and the exploration of the factors causing the differences. Korean J Local Gov Stud 2012;15:31-57 (Korean).

[5] Seok HS, Kang SH. A study on the regional variation factor of hypertension prevalence. Health Soc Welf Rev 2013;33:210-236 (Korean).

https://doi.org/10.4178/epih.e2017025

[6] Kim YM, Cho DG, Kang SH. An empirical analysis on geographic variations in the prevalence of diabetes. Health Soc Welf Rev 2014;34:82-105 (Korean).

[7] Kim Y, Cho D, Hong S, Kim E, Kang S. Analysis on geographical variations of the prevalence of hypertension using multi-year data. J Korean Geogr Soc 2014;49:935-948 (Korean).

[8] Kim YM, Kang SH. Changes and determinants affecting on geographic variations in health behavior, prevalence of hypertension and diabetes in Korean. J Digit Converg 2015;13:241-254 (Korean).

[9] Kim YM, Kang SH. Convergence analysis of determinants affecting on geographic variations in the prevalence of arthritis in Korean women using data mining. J Digit Converg 2015;13:277-288 (Korean).

[10] Lee JH, Park KS, Kim RB, Kim BJ, Chun JH. The influence of individual-level social capital on depression. J Agric Med Community Health 2011;36:73-86 (Korean).

[11] Min SY. Depression, self-esteem, and quality of life in a community population. J Korean Acad Psychiatr Ment Health Nurs 2010; 19:1-10 (Korean).

[12] Nam IS, Hyun DW. An analysis of gender differentials in depression: the impact of inequality. Korean Soc Secur Stud 2014;30:143163 (Korean).

[13] Wells KB, Stewart A, Hays RD, Burnam MA, Rogers W, Daniels M, et al. The functioning and well-being of depressed patients. Results from the Medical Outcomes Study. JAMA 1989;262:914919.

[14] World Health Organization. The world health report 2001- mental health: new

understanding, new hope [cited 2017 Jul 31]. Available from: http://www.who.int/whr/2001/en/.

[15] Murray CJ, Vos T, Lozano R, Naghavi M, Flaxman AD, Michaud C, et al. Disability-adjusted life years (DALYs) for 291 diseases and injuries in 21 regions, 1990-2010: a systematic analysis for the Global Burden of Disease Study 2010. Lancet 2012;380:2197-2223.

[16] Joormann J, Tanovic E. Cognitive vulnerability to depression: examining cognitive control and emotion regulation. Curr Opin Psychol 2015;4:86-92.

[17] Østergaard D, Dalton SO, Bidstrup PE, Poulsen AH, Frederiksen K, Eplov LF, et al. Mental vulnerability as a risk factor for depression: a prospective cohort study in Denmark. Int J Soc Psychiatry 2012;58:306-314.

[18] Kim W, Hwang TY, Ham BJ, Lee JS, Choi BH, Kim SJ, et al. The impact of major depressive disorder on productivity in workers: a preliminary study using WHO-HPQ (Health and Work Performance Questionnaire). J Korean Neuropsychiatr Assoc 2007;46: 587-595 (Korean).

[19] Rho BI, Kwak HK. A study on the effect of neighborhood-level contextual characteristics on mental health of community residents. Health Med Sociol 2005;17:5-31 (Korean).

[20] Choi MY. An empirical study of the effects of neighborhoods and social capital on elderly people depression. Korean J Soc Welf Res 2008;18:25-46 (Korean).

[21] De Silva MJ, Huttly SR, Harpham T, Kenward MG. Social capital and mental health: a comparative analysis of four low income countries. Soc Sci Med 2007;64:5-20.

[22] Wang H, Schlesinger M, Wang H, Hsiao WC. The flip-side of social capital: the distinctive influences of trust and mistrust on health in rural China. Soc Sci Med 2009;68:133-142.

[23] Kang DA. A study on factors influencing the depression of the elderly in rural [dissertation]. Daejeon: Mokwon University; 2005 (Korean).

[24] Jung HS. A study on the influencing factors of depression in the elderly [dissertation]. Gongju: Kongju National University; 2007 (Korean).

[25] Kim TS, Kim DJ. The association between smoking and depression. Korean J Psychopharmacol 2007;18:393-398 (Korean).

[26] Korea Centers for Disease Control and Prevention. 2008-2014 Community health statistics to look at a glance; 2015 [cited 2017 Jul 25]. Available from: https://chs.cdc.go.kr/chs/sub03/sub03_03_ 01.do (Korean).

[27] American Psychiatric Association. What Is depression? [cited 2017 Sep 3]. Available from: https://www.psychiatry.org/patients-families/depression/what-is-depression.

[28] Noh SC, Lee HY. Advanced statistical analysis methodology. Seoul: Moonwoosa; 2013, p. 619-625 (Korean).

[29] Hammen C. Stress and depression. Annu Rev Clin Psychol 2005; 1:293-319.

[30] Burke HM, Davis MC, Otte C, Mohr DC. Depression and cortisol responses to psychological stress: a meta-analysis. Psychoneuroendocrinology 2005;30:846-856.

[31] Korea Centers for Disease Control and Prevention. The factbook on chronic disease; 2015 [cited 2017 May 30]. Available from:

http://www.cdc.go.kr/CDC/notice/CdcKrIntro0504.jsp?menuIds= HO ME001-MNU1154-MNU0005-MNU2572-MNU0110&cid =65024 (Korean).

[32] Thune JJ, Signorovitch JE, Kober L, McMurray JJ, Swedberg K, Rouleau J, et al. Predictors and prognostic impact of recurrent myocardial infarction in patients with left ventricular dysfunction, heart failure, or both following a first myocardial infarction. Eur J Heart Fail 2011;13:148-153.

[33] Park JH, Bae SH. A systematic review of psychological distress as a risk factor for recurrent cardiac events in patients with coronary artery disease. J Korean Acad Nurs 2011;41:704-714 (Korean).

[34] Lee HR. Atopic dermatitis. J Korean Pediatr Soc 2000;43:1161-1167 (Korean).

[35] Lee KS, Oh EY, Park JH, Hong CH, Lee SY. Comparison of child behavior checklist between allergic and non-allergic patients. J Asthma Allergy Clin Immunol 2001;21:178-186 (Korean).

[36] Lee SA, Lee GM. A study on the major factors influencing the depression among the elderly in rural area. J Korea Gerontol Soc 2002;22:209-226 (Korean).

[37] Jee Y, Goo C. The Differences of abilities to physical activity, social networks, depression degrees of elderly between residing rural and urban. J Coach Dev 2007;9:425-439 (Korean).

Volume: 39, Article ID: e2017025

https://doi.org/10.4178/epih.e2017025

Table 1. Descriptive statistics of indicators related to depression

Indicators	Mean	Min	Max	Std.	Extremal Quotient
rate of experiencing depressive symptoms	5.07	1.05	10.80	2.13	10.29
rate of exposure to secondhand smoke at home	11.21	4.30	22.00	2.99	5.12
annual rate of drinking	73.53	53.25	82.75	5.93	1.55
monthly rate of drinking	58.18	41.45	65.75	4.40	1.59
age at drinking onset (years)	21.23	20.05	22.65	0.49	1.13
rate of walking	40.55	15.25	69.30	10.44	4.54
rate of performing vigorous exercise	14.46	7.35	31.80	3.92	4.33
rate of performing muscle strengthening exercise	18.28	6.15	33.15	3.96	5.39
rate of performing moderate to high intensity physical activities	13.49	3.60	54.45	7.80	15.13
level of fruit consumption	42.43	20.95	69.00	7.68	3.29
weekly breakfast intake frequency of 1-2 times	5.94	1.85	9.50	1.57	5.14
rate of consulting a psychologist due to stress	5.48	0.80	12.65	2.00	15.81
rate of the perceived stress level	27.11	15.05	35.55	3.89	2.36
rate of sleeping less than 6 hours per day	42.89	25.30	54.05	6.17	2.14
rate of lifetime diagnosis of hypertension	14.89	10.95	19.50	1.66	1.78
rate of lifetime diagnosis of diabetes	5.69	4.00	8.20	0.75	2.05
rate of lifetime diagnosis of cerebral infarction (stroke)	1.08	0.50	2.20	0.29	4.40
rate of lifetime diagnosis of myocardial infarction	0.79	0.25	1.40	0.23	5.60
rate of lifetime diagnosis of atopic dermatitis	2.88	0.30	6.15	1.02	20.50
rate of lifetime diagnosis of allergic rhinitis	12.88	1.85	21.25	3.70	11.49
rate of lifetime diagnosis of asthma	2.10	0.80	3.80	0.59	4.75
rate of lifetime diagnosis of angina	1.06	0.40	1.95	0.28	4.88
rate of not receiving necessary medical services	13.61	2.70	25.30	3.41	9.37

Volume: 39, Article ID: e2017025

https://doi.org/10.4178/epih.e2017025

Contact with social networks (neighbors) less than once per month	33.31	0	68.70	17.61	0.00
Leisure environment	29.30	14.30	61.35	7.47	4.29
Religious environment	26.13	11.15	44.10	6.41	3.96
Social environment	56.77	40.00	77.85	7.44	1.95
					Continued
Public transit environment	65.05	29.00	93.10	14.45	3.21
Supportive environment	51.87	15.40	95.65	22.90	6.21
Trust environment	66.41	39.65	97.90	11.49	2.47
Safety environment	75.35	48.50	95.05	9.56	1.96
Healthcare environment	62.82	23.35	90.40	13.33	3.87
Natural environment	80.30	36.60	97.80	11.51	2.67

Table 2. Correlation between indicators and depression and spatial autocorrelation

Indicators	Correlation	p value	Moran's I	Z score	p value
rate of experiencing depressive symptoms	-	-	0.34	27.85	<.0001
rate of lifetime diagnosis of depression	-	-	0.06	5.47	<.0001
rate of exposure to secondhand smoke at home	0.24	0.0001	0.03	3.18	0.001886
rate of exposure to secondhand smoke in public	0.41	<.0001	0.20	16.736188	<.0001
annual level of drinking	0.51	<.0001	0.33	26.96	<.0001
monthly level of drinking	0.46	<.0001	0.32	26.0861	<.0001
age at drinking onset (years)	-0.49	<.0001	0.36	29.459516	<.0001
rate of walking	0.20	0.0011	0.37	30.2492	<.0001
rate of performing rigorous physical activities	-0.29	<.0001	0.06	5.39408	<.0001
rate of performing muscle strengthening exercise	0.55	<.0001	0.43	34.634041	<.0001
rate of performing moderate to high intensity physical activities	-0.35	<.0001	0.28	22.627235	<.0001
level of fruit consumption	0.33	<.0001	0.28	23.053	<.0001
weekly breakfast intake frequency of 1-2 times	0.59	<.0001	0.37	30.087411	<.0001
rate of consulting a psychologist due to stress	0.45	<.0001	0.10	8.316251	<.0001
perceived stress level	0.57	<.0001	0.32	25.957428	<.0001
rate of sleeping less than 6 hours per day	0.58	<.0001	0.59	47.2999	<.0001
rate of lifetime diagnosis of hypertension	0.35	<.0001	0.59	47.2999	<.0001
rate of lifetime diagnosis of diabetes	0.21	0.0007	0.06	4.983583	<.0001
rate of lifetime diagnosis of cerebral infarction (stroke)	0.20	0.0011	0.03	3.045525	0.002323
rate of lifetime diagnosis of myocardial infarction	0.37	<.0001	0.04	3.827217	0.00013
rate of lifetime diagnosis of atopic dermatitis	0.59	<.0001	0.54	43.125389	<.0001
				Continued	
rate of lifetime diagnosis of allergic rhinitis	0.65	<.0001	0.42	34.144919	<.0001

Volume: 39, Article ID: e2017025

https://doi.org/10.4178/epih.e2017025

rate of lifetime diagnosis of asthma	0.41	<.0001	0.12	10.257864	<.0001
rate of lifetime diagnosis of angina	0.23	0.0002	0.07	5.809221	<.0001
rate of not receiving necessary medical services	0.40	<.0001	0.00	0.378656	0.704943
Contacting social networks (neighbors) less than once per month	0.47	<.0001	0.51	40.656439	<.0001
Leisure environment	0.41	<.0001	0.34	27.468532	<.0001
Religious environment	0.24	0.0001	0.44	27.468532	<.0001
Social environment	-0.47	<.0001	0.39	31.122194	<.0001
Public transit environment	0.28	<.0001	0.41	33.377054	<.0001
Supportive environment	-0.47	<.0001	0.49	39.614313	<.0001
Trust environment	-0.55	<.0001	0.50	40.423398	<.0001
Safety environment	-0.47	<.0001	0.31	25.416693	<.0001
Healthcare environment	0.14	0.00253	0.22	18.156994	<.0001
Natural environment	-0.40	<.0001	0.33	26.397	<.0001

Volume: 39, Article ID: e2017025

https://doi.org/10.4178/epih.e2017025

Table 3. Association between community characteristics and depression

**model**	**GWR (N=251)**				**OLS (N=251)**	
	Mean	Min	Max	Std.	B	p value
Constant	-1.88	-3.31	-0.42	1.04	-1.98	0.0011
The rate of lifetime diagnosis of myocardial infarction	1.21	0.48	1.59	0.34	1.12	0.0021
The rate of lifetime diagnosis of allergic rhinitis	0.17	0.13	0.23	0.03	0.18	<.0001
The weekly breakfast intake frequency of 1-2 times	0.26	0.17	0.35	0.05	0.24	0.0005
The perceived stress level	0.10	0.07	0.13	0.02	0.10	<.0001
Public transit environment	-0.01	-0.02	0.002	0.01	-0.01	0.0062
R^2	0.58				0.54	
R^2_{adj}	0.55				0.53	
AIC	808.714				815.417	
Normality	Jarque-Bera				7.0584*	
Homoscedasticity	Koenker-Bassett				9.2515	

[1]GWR = Geographically Weighted Regression

[2]OLS = Ordinary least square

[3]AIC = Akaike's Information Criterion

*p value < 0.05

Volume: 39, Article ID: e2017025

https://doi.org/10.4178/epih.e2017025

Table 4. Comparative analysis of models

Model	Comparative analysis	model 1[1)]	model 2[2)]	model 3[3)]	model 4[4)]
GWR					
	R^2	0.53	0.52	0.35	0.58
	R^2_{adj}	0.50	0.50	0.33	0.55
	AIC	839.36	833.14	905.07	808.71
OLS					
	R^2	0.48	0.50	0.31	0.54
	R^2_{adj}	0.47	0.49	0.35	0.53
	AIC	847.83	836.32	913.91	815.42
	Koenker Statistics	19.0774*	5.7374	5.5818	7.0584*
	Jarqure-Bera Statistics	4.3914	5.2279	6.5397	9.2515

[1)]A model consisting of health behavior indicators

[2)]A model consisting of disease indicators

[3)]A model consisting of social-physical environment indicators

[4)]final model

*p value < 0.05

[5]GWR = Geographically Weighted Regression

[6]OLS = Ordinary least square

[7]AIC = Akaike's Information Criterion

Volume: 39, Article ID: e2017025

https://doi.org/10.4178/epih.e2017025

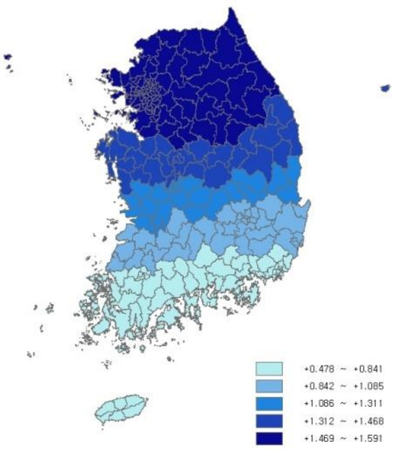

(A)

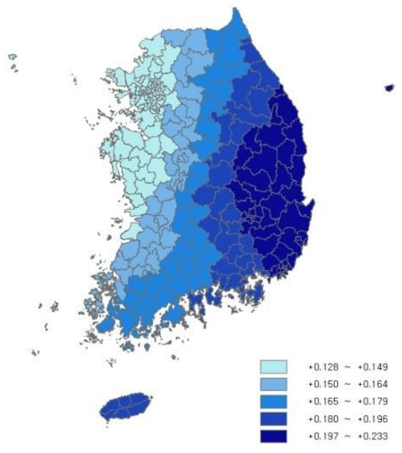

(B)

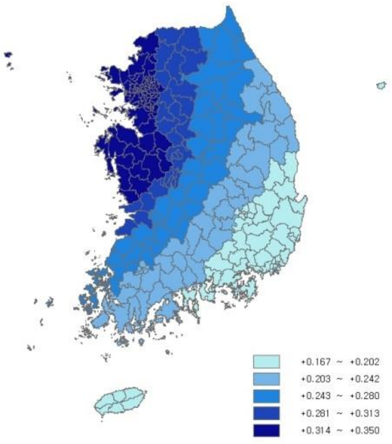

(C)

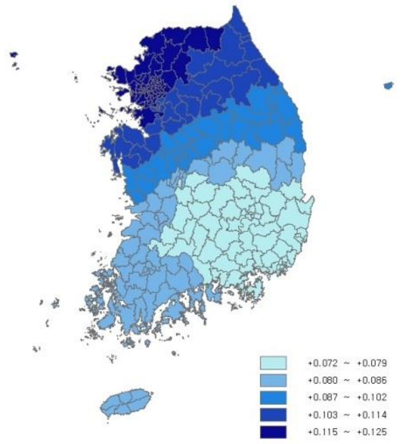

(D)

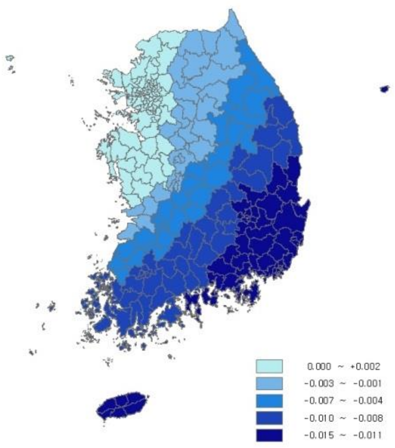

(E)

Figure 1. Regression coefficient based on geographically weighted regression (A) rate of lifetime diagnosis of myocardial infarction, (B) rate of lifetime diagnosis of allergic rhinitis, (C) weekly breakfast intake frequency of 1-2 times, (D) perceived stress level, and (E) public transit environment.

Volume: 39, Article ID: e2017025

https://doi.org/10.4178/epih.e2017025

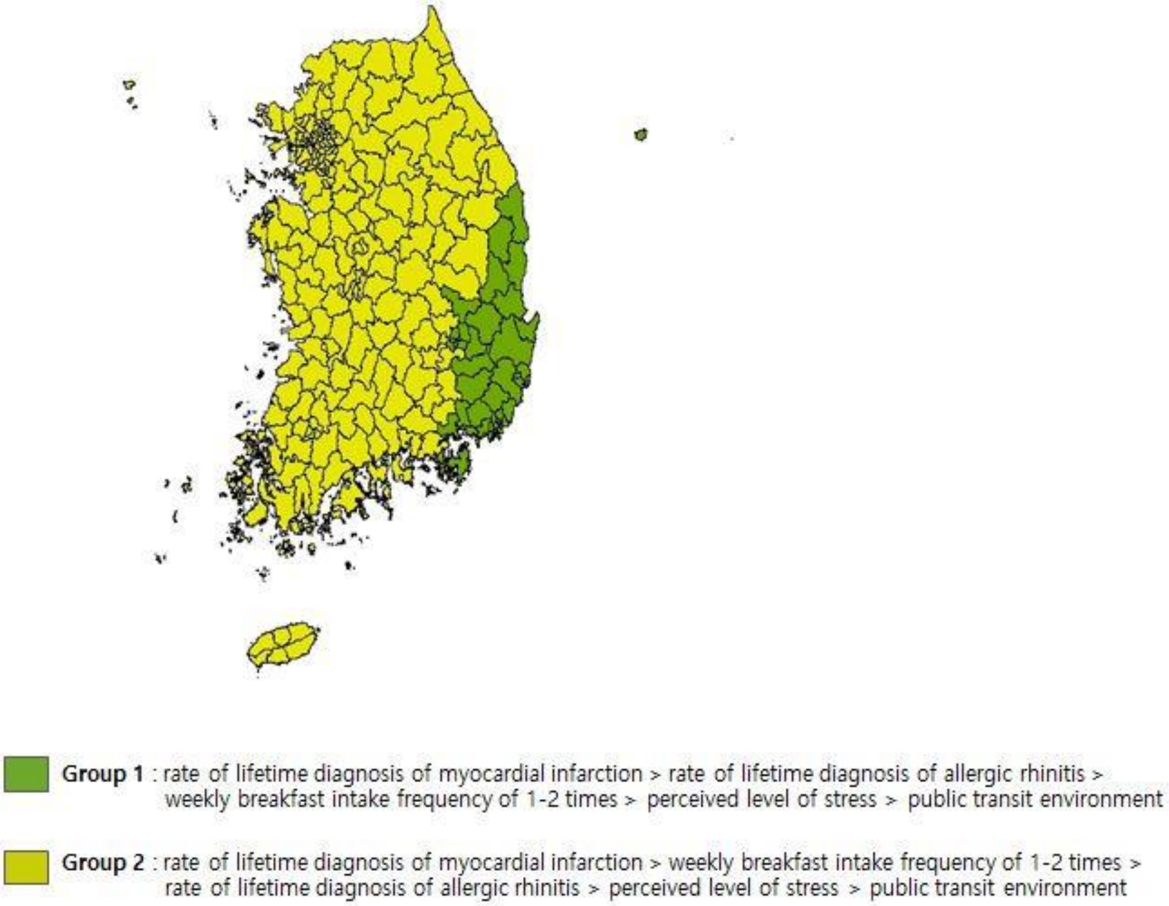

Figure 2. Classification based on the ranking of influence factors.

Volume: 39, Article ID: e2017025

https://doi.org/10.4178/epih.e2017025

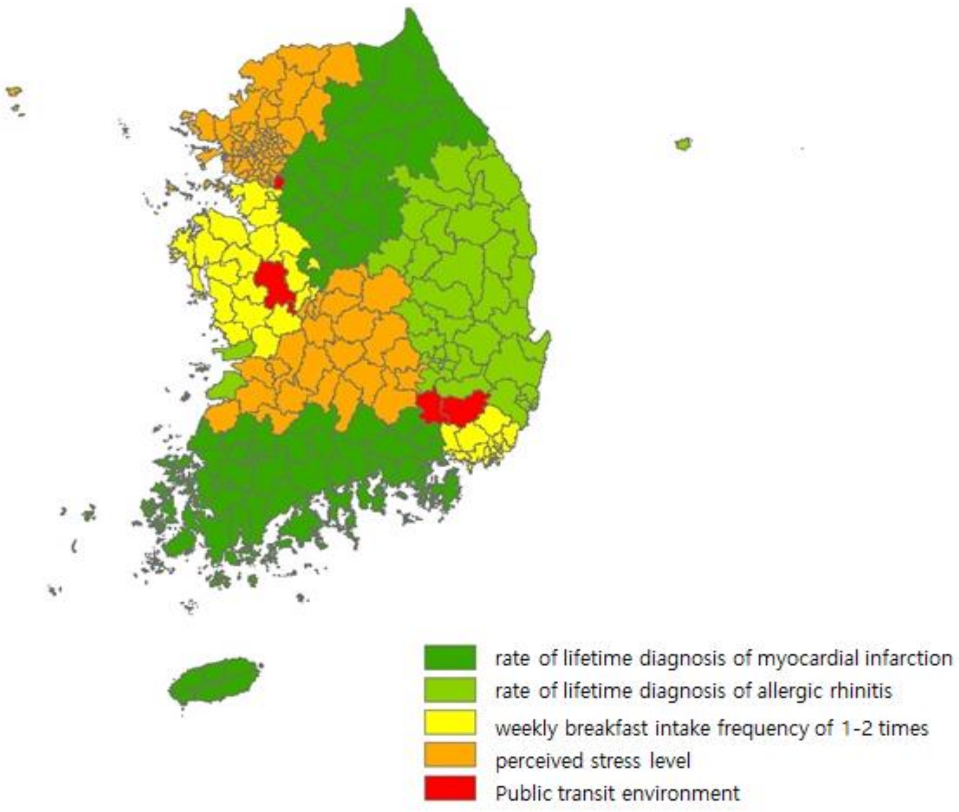

Figure 3. Classification based on the relative ranking of influence factors.
